# The correlation between humoral immune responses and severity of clinical symptoms in COVID-19 patients

**DOI:** 10.1017/S0950268823001437

**Published:** 2023-09-11

**Authors:** Shadi Akbarian, Mehdi Sheikhi, Parichehr Khedri, Narges Baharifar, Fatemeh Khalaf Shamsabadi, Mohammad Reza Davidi, Hossein Ali Khazaei, Hamidali Assarian, Abdolkarim Sheikhi

**Affiliations:** 1 Department of Microbiology, Borujerd Branch, Islamic Azad University, Borujerd, Iran; 2 Faculty of Medicine, Kazeroon Azad University, Kazeroon, Iran; 3 Department of Immunology, Faculty of Medicine, Dezful University of Medical Sciences, Dezful, Iran; 4 Department of Immunology, Faculty of Medicine, Zahedan University of Medical Sciences, Zahedan, Iran; 5 Department of Microbiology, Dr. Assarian Pathobiology Lab, Dezful, Iran

**Keywords:** COVID-19, clinical symptoms, IgG, IgM, immune system

## Abstract

The SARS-CoV-2 pandemic persists with global repercussions. Initial COVID-19 symptoms encompass pneumonia, fever, myalgia, and fatigue. The human immune system produces IgM and IgG antibodies in response to SARS-CoV-2. Despite previous research, a comprehensive understanding of the interplay between clinical manifestations and humoral immune responses remains elusive. This study aims to scrutinize this association. 134 COVID-19 patients were enrolled, and stratified into mild, moderate, and severe symptom groups. Serum IgM and IgG levels were assessed thrice at one-month intervals using ELISA. The findings reveal significant elevation in serum IgG levels in moderate compared to mild cases (*P* < 0.001). Additionally, IgG production was significantly heightened in severe cases compared to both mild (*P* < 0.0001) and moderate (*P* < 0.05) groups. IgM and IgG levels peaked initially and diminished over time. While anti-SARS-CoV-2 antibodies are expected to confer protection, the direct correlation between IgG levels and symptom severity may arise from delayed immune activation, resulting in an intense antibody response in severe cases. Given evidence linking delayed immune function with a dysregulated innate immune response, comprehensive data collection should encompass not only serum IgG and IgM, but also early measurement of type I interferons at symptom onset. This could provide a more thorough understanding of COVID-19 progression.

## Introduction

Severe acute respiratory syndrome coronavirus 2 (SARS‑CoV‑2) is a novel beta-coronavirus responsible for the coronavirus disease 2019 (COVID-19) pandemic which first occurred in patients with pneumonia symptoms in Wuhan, China in December 2019 [1]. Coronaviruses belong to the family of *Coronaviridae*, the order *Nidovirales*, and the genus *Coronavirus.* The family contains two subfamilies, *Coronavirinae* and *Torovirinae.* *Coronavirinae* are categorized into four important genera that include *Alphacoronavirus*, *Betacoronavirus*, *Gammacoronavirus*, and *Deltacoronavirus.* With the discovery of the genomic sequence of SARS‑CoV‑2, this virus has also been placed in the *Betacoronavirus* genus [2]. SARS-CoV-2 is a positive-sense single-stranded enveloped RNA coronavirus with one of the largest known RNA viral genomes (∼29.8 kb). The genome of this virus is strikingly similar to those of other coronaviruses, especially the SARS-CoV and bat coronavirus. There are various proteins, known as antigens, in the structure of this virus, including spike (S), membrane (M), envelope (E), and nucleocapsid (N) antigens [3–4].

An efficient immune response is essential to control and eradicate this coronavirus. In COVID-19 infection, any immune system dysfunction can lead to morbidity and mortality. A better understanding of how the immune system works against COVID-19 can be very effective in controlling the disease. Similar to other viruses, the immune system can detect coronavirus via innate immune receptors such as Toll-like receptor (TLR), RIG-I-like receptor (RLR), NOD-like receptor (NLR), and C-type lectin-like receptors. Type I interferons (INFs) are at the forefront of defence against viruses [5]. Studies have demonstrated that even though SARS-CoV, SARS-CoV-2, and other coronaviruses are sensitive to IFN-α and IFN-β, they remain pathogenic. These viruses escape the host immune system using various methods such as inhibiting the JAK-STAT pathway downstream of type I interferons [6]. On the other hand, the virus interferes with the differentiation and function of dendritic cells, thereby affecting specific immune responses [5]. CD4^+^ T cells play an important role in immunity against SARS-CoV-2 by stimulating the production of virus-specific antibodies by B cells. CD8+ T cells are cytotoxic lymphocytes and manage to kill virus-infected cells. The antibody responses in the body also include a dynamic and complex set of antibodies that target different antigens on the surface of SARS‑CoV‑2. The virus uses its surface proteins as an adhesion factor to enter host cells through a special receptor called angiotensin-converting enzyme 2 (ACE-2) [7]. Studies have shown that in the primary immune response, IgM antibodies are produced in low quantities. In contrast, IgG production is delayed, but due to the creation of immune memory, the production of this class of antibodies is higher. These antibodies remain in the serum for a longer period, even after the infection is resolved. Therefore, the detection of IgM in a patient’s serum could be immunological evidence of a recent infection, whereas the detection of IgG in the serum of a person who has no clinical symptoms often indicates a previous infection.

COVID-19 causes a variety of symptoms, especially in the respiratory system. Clinical symptoms vary from asymptomatic to acute respiratory syndrome and dysfunction of several organs, but common clinical symptoms include fever, cough, sore throat, headache, fatigue, shortness of breath, and conjunctivitis. Some patients may not have obvious symptoms, so a computerized tomography (CT) scan may be a suitable approach to diagnose the disease in early stages when the clinical symptoms are nonspecific or rare. In 2020, Zhou et al. reported bilateral changes in the lungs of most COVID-19 patients. These bilateral changes were evident on chest X-ray or CT scans [8–10].

Considering that the humoral immune response, especially specific antibodies, plays a prominent role in neutralizing viruses, the purpose of this study is to investigate the relationship between the severity of clinical symptoms and the level of specific antibodies in the serum of COVID-19 patients.

## Materials and methods

### Study design and patient enrolment

**Figure 1.**
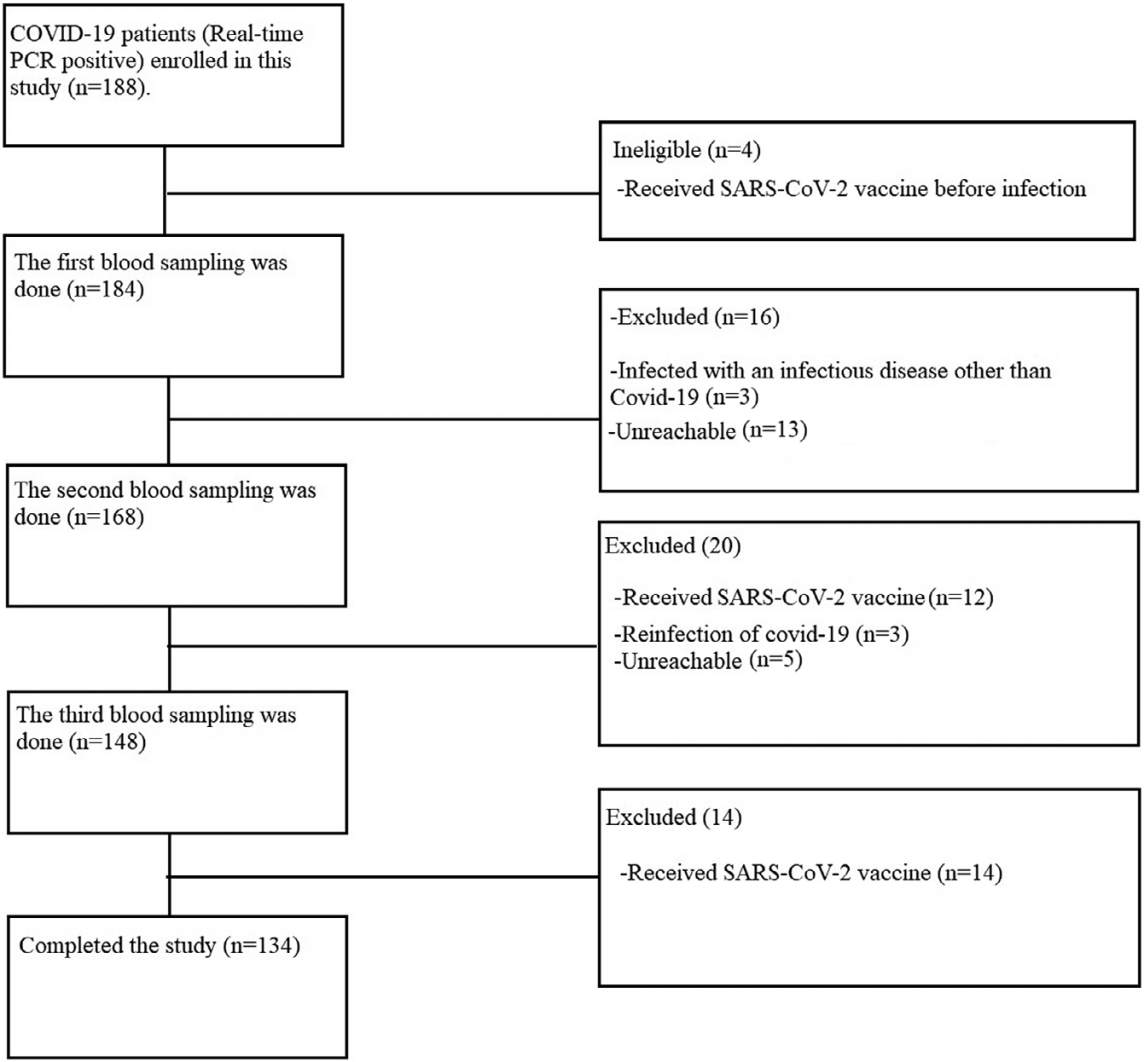
Study flowchart.

This cross-sectional study was conducted between 25 May 2020, and 19 October 2020. A total of 188 patients with COVID-19 were admitted to Ganjavian Hospital in Dezful; of 188 patients, only 134 patients were finally included in the study (Figure 1).

After obtaining informed consent from the participants, they entered the study. An amount of 3 ml of blood sample was taken from the patients on three occasions, one month apart. The first blood sample was taken at least 25 days after the onset of symptoms. The second and third samples were one month and two months after the first sampling, respectively.

Study participants were adults, male and female, unvaccinated against COVID-19 with a positive quantitative reverse transcription polymerase chain reaction (RT-qPCR) test.

The patients were subdivided based on the type of and the severity and duration of the clinical symptoms, duration of hospitalization, and oxygen requirement. A severity evaluation occurred at the time of the first blood sample based on the patient’s registered file at the hospital.

### Detection of IgG and IgM against SARS-CoV-2

Blood samples were used to evaluate IgM and IgG using an enzyme-linked immunosorbent assay (ELISA) kit (Euroimmun, Lubeck, Germany). The commercial anti-SARS-CoV-2 S1 ELISA IgG kit and nucleocapsid protein-specific IgM kit were used. Results were analyzed and interpreted according to the manufacturer’s instructions.

### Statistical analysis

Results were expressed as mean ± SD. Differences in mean values between groups were analyzed with the Wilcoxon signed-rank, Mann–Whitney U, and Kruskal–Wallis tests to reveal significant differences between the IgG and IgM levels in patients’ serum. Differences were considered to be significant at *P* < 0.05. Statistical calculations were performed with SPSS 19 (SPSS Inc., Chicago, IL).

## Results

### Grouping samples

The patients were divided into 3 groups based on their symptoms (Table 1). Common symptoms included headache and anosmia, observed in almost all patients. As we go from the first to the third group, the condition of the patient with SARS‑CoV‑2 becomes worse and the clinical symptoms become more severe. More than 50% of group C patients were hospitalized, while only 16% of group A patients were hospitalized. Group C patients were overweight and had underlying health-related conditions such as diabetes or heart disease. According to the results 5.9%, 11.9%, and 29.7% of patients from groups A, B, and C, respectively, required respiratory support and oxygen therapy. The difference between the ages of groups A, B, and C was not significant.

**Table 1.** Grouping of COVID-19 patients based on symptoms

			Gender				
Groups	*n*	Age (Average ± SD)	Male	Female	Respiratory support %	Hospitalization %	Symptoms
Group A (mild symptoms): flu-like without fever	51	36.6 ± 9.7	11 (22%)	40 (78%)	5.9	16	Headache, anosmia, muscle aches, cough, sore throat, no fever, hoarseness, loss of appetite
Group B (moderate symptoms): flu-like with fever, gastrointestinal symptoms, fatigue	38	37.7 ± 9.8	8 (19%)	30 (79%)	13.2%	26.3	Headache, anosmia, loss of appetite, diarrhoea, sore throat, cough, fever, hoarseness, chest pain, fatigue
Group C (severe symptoms): flu-like with fever, dizziness, severe abdominal and respiratory symptoms	45	37.4 ± 9.5	9 (20%)	36 (80%)	31.1%	51.1	Headache, anosmia, loss of appetite, cough, fever, hoarseness, sore throat, chest pain, fatigue, dizziness, muscle aches, confusion, dyspnoea, diarrhoea, abdominal pain

### IgG and IgM levels in COVID-19 patients with mild (A), moderate (B), and severe (C) symptoms

**Figure 2.**
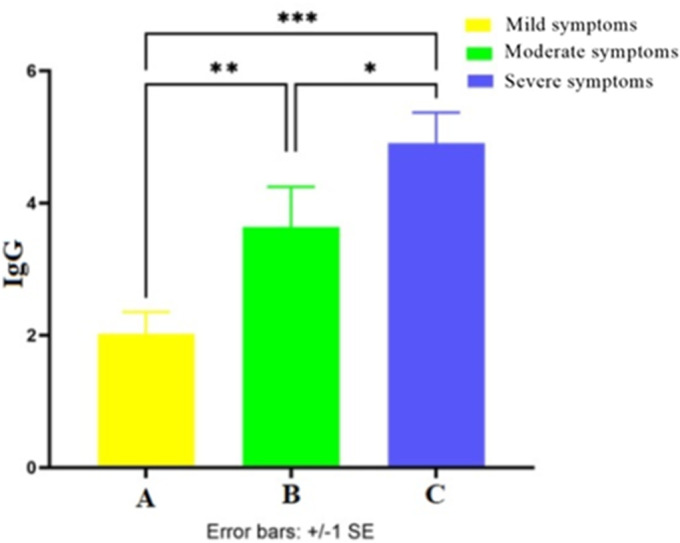
The average of IgG level in the mixed three (first, second, and third) samplings of groups A, B, and C. *P < 0.05, **P < 0.001, ***P < 0.0001.

As indicated in Figure 2, COVID-19 patients with severe symptoms (group C) had the highest serum IgG level and the mild group (group A) had the lowest IgG level. A statistical comparison of groups A, B, and C revealed that the serum IgG level was significantly higher in group B in comparison with group A (*P* = 0.0009). IgG production was also significantly higher in group C in comparison with groups A and B (*P* = 0.00009, *P* = 0.03 respectively).

**Figure 3.**
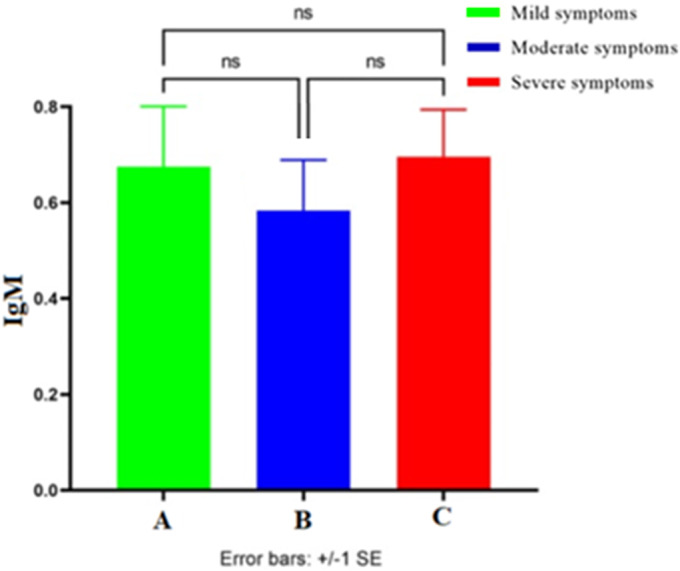
The average of IgM level in the mixed three (first, second, and third) samplings of groups A, B, and C. ns, nonsignificant.

As Figure 3 indicates the difference between the serum level of IgM in groups A, B, and C is not significant.

### Comparing IgG levels in COVID-19 patients with mild (A), moderate (B), and severe (C) symptoms based on the sampling time interval from the onset of the disease

The time interval of the first sampling was at least 25 (34.2±7.8) days after the onset of the disease, and of the second and third sampling were one month and two months from the first sampling, respectively.

**Figure 4.**
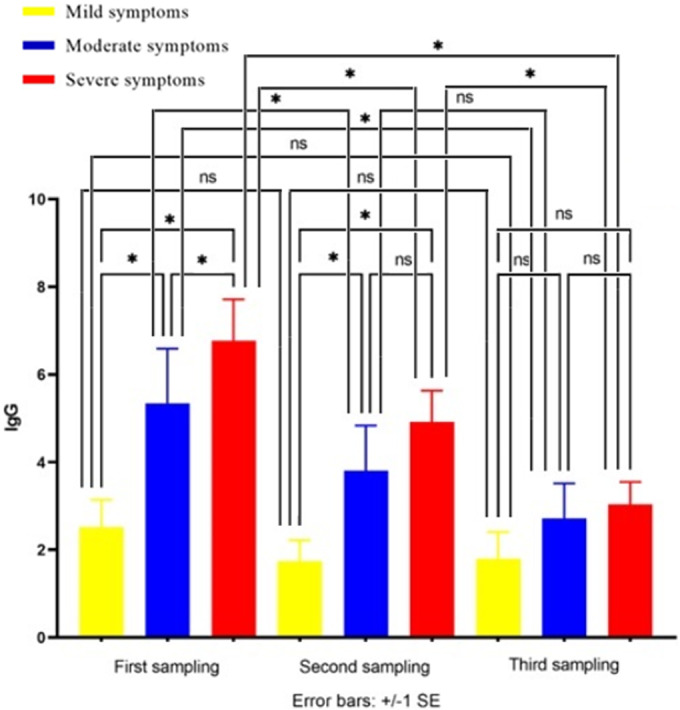
The IgG level in first, second, and third serum samples of groups A, B, and C. ns, nonsignificant, *P < 0.05.

As indicated in Figure 4, in the first sampling, the difference in IgG level between groups A (mild), B (moderate), and C (severe) was significant while in the second sampling, the difference in IgG level between groups B and C was not significant. Furthermore, in the third sampling, the difference in IgG level between none of the groups was significant. In other words, the earlier we take the samples, the difference between the IgG levels of groups A, B, and C becomes more significant.

In the severe group (group C) and the moderate group (group B), the IgG level at the first sampling was significantly higher than the second sampling, and the IgG level in the second sampling was significantly higher than the third sampling, while in the mild group (group A), there were no significant differences in the first, second, and third samplings. In other words, the decrease of IgG levels from the first to second, second to third, and first to third samples in group A was not significant (Figure 4).

**Figure 5.**
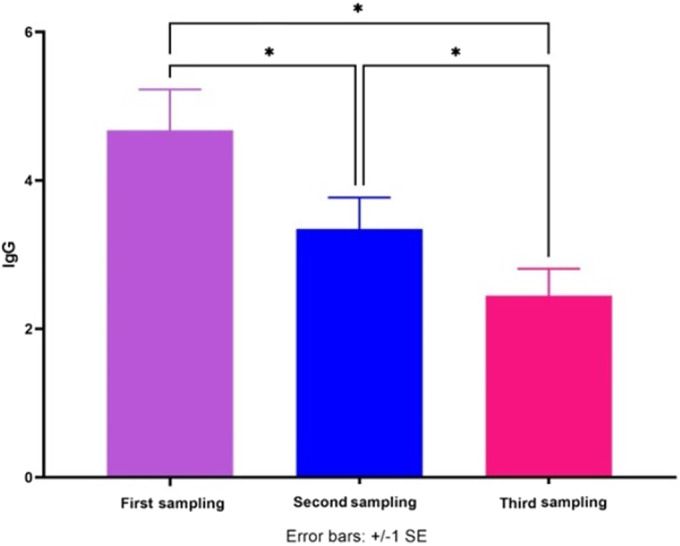
The average of IgG secretion of all patients (A, B, and C groups) in the first, second, and third samples. *P < 0.05.

As indicated in Figure 5, the average of IgG level of all patients (A, B, and C groups) in the third samples was significantly lower than in the first and second samples. Also, the average of IgG level of all patients in the second samples was significantly lower than in the first samples.

### Comparing the IgM level in COVID-19 patients with mild (A), moderate (B), and severe (C) symptoms based on the sampling time interval from the onset of the disease

**Figure 6.**
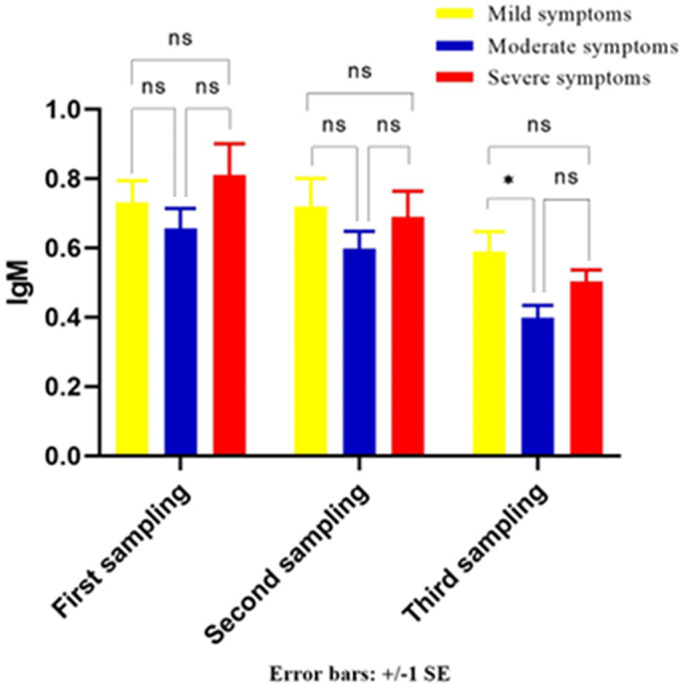
The IgM level in first, second, and third serum samples of groups A (mild), B (moderate), and C (severe). ns, nonsignificant, *P < 0.05.

As indicated in Figure 6, the difference between secreted IgM levels in the serum of groups A (mild), B (moderate), and C (severe) in the first and second samplings was not significant. Just the difference between the secreted IgM levels in the serum of groups A and B at third sampling is statistically significant.

**Figure 7.**
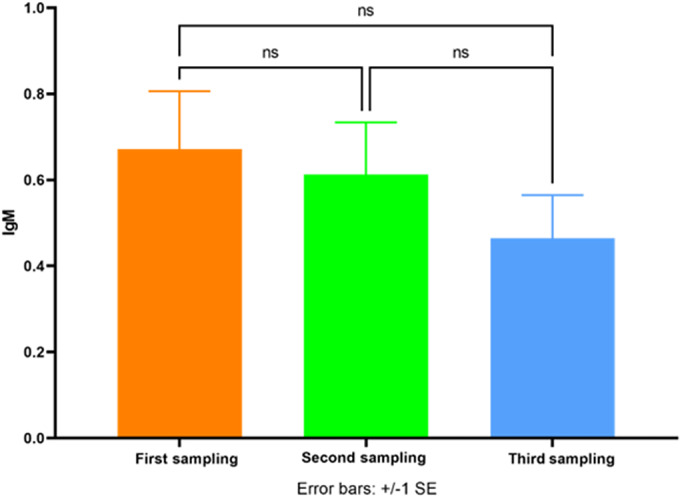
The average IgM level in all patients (A, B, and C groups) in the first, second, and third samples. ns, nonsignificant.

As Figure 7 indicates, the average IgM level in all COVID-19 patients non-significantly decreased in the third samples in comparison with the second samples (*P* = 0.6556). The IgM level also decreased in the second samples in comparison with the first samples (*P* = 0.9347) which was not statistically significant.

## Discussion

This study aims to evaluate the level of antibodies and describe clinical manifestations and the relationship between antibody levels and the severity of disease symptoms in COVID-19 patients.

COVID-19 is a life-threatening infectious disease and clinical symptoms include fever, cough, shortness of breath, loss of sense of smell and taste, and fatigue. The disease is mostly transmitted through breathing and close contact and is a major threat to global health. The incidence and severity of the disease depend on the interaction between the virus and the human immune system. The state of the human immune system, age, gender, physical condition, nutrition, hygiene, homeostasis (between the immune, nervous, and endocrine systems), virus mutations, and the number of virus particles entering the human body are all important factors involved in the emergence, severity, and relapse of the disease [11].

It is widely known that once a virus enters the body, the innate immune system detects it and immediately activates the specific immune system. Innate immune system cells, such as macrophages, take up viral particles and, after processing, present them to specific lymphocytes. Then, IgM antibodies are produced first, which are low in quantity and quality, and their production time is short. Then, as the immune response progresses and receives appropriate signals, B lymphocytes do a class switch and produce mainly IgG antibodies, which will remain in the serum for a longer period, even after the infection is resolved [12].

Various studies have demonstrated that seroconversion of COVID-19 is very similar to other acute viral infections meaning that as the IgM level approaches its maximum concentration the IgG level begins to increase. However, it has been reported that the increase in IgM and IgG titres against SARS-CoV-2 is slower than other respiratory system viruses [13].

Some studies indicate that anti-SARS-CoV-2 IgM antibody appears about 5 days after the onset of symptoms and its titre increases rapidly, and the maximum anti-virus IgM titre is observed on days 18-22 after which the IgM level decreases. Anti-SARS-CoV-2 IgG also appears about 9 days after the onset of symptoms and its titre increases rapidly and the maximum anti-SARS-CoV-2 IgG titre is observed in 24 days and then remained high for a long time. However, for both types of immunoglobulins, patients with severe forms of disease showed higher titres of antibodies compared to patients with mild forms of disease at all times of antibody level assessment [14].

In this study, blood samples were taken from 134 patients with COVID-19 on three occasions, one month apart. IgG and IgM levels were measured and patients were divided into 3 groups based on the severity of clinical COVID-19 symptoms. The more we move from group A to group C, the worse the condition of the patient with SARS-CoV-2 becomes. As indicated in the results section, more than 50% of group C patients were hospitalized while only 16% of group A patients were hospitalized. Most commonly, patients in groups B and C were less able-bodied and overweight and had underlying health-related conditions such as diabetes or heart disease.

In this study, titres of IgG and IgM were assessed on three occasions, one month apart. As the results indicated, the IgG level significantly decreased over time (*P* < 0.05), but the reduction of IgM over time was not statistically significant (*P* > 0.05) which can be due to the small number of samples.

In another comparison, the levels of IgM and IgG were evaluated based on the severity of symptoms (severe, moderate, and mild) at different time points from the onset of the disease.

IgM is the first antibody that is produced and has a lower affinity than IgG. The increased level of IgM is an indicator of disease onset and its level may vary from person to person. IgM levels were expected to decrease over time in patients with different symptom levels (severe, moderate, and mild), but no significant changes in IgM levels were observed in this study. This may be due to the small number of samples. Moreover, when measuring IgM using ELISA-based methods, if the amount of IgG in the serum is higher than IgM (in this study, we measured IgM and IgG at least 25 days after the onset of the disease), then the amount of IgG is so higher compared to IgM; therefore, IgG (because of higher affinity) occupies the epitopes and no longer allows IgM to bind to the epitopes. Since the level of IgG at the second and third samples is decreased, the amount of measured IgM in the third samples is closer to the real titre. Thus, the amount of IgM in the first samples compared to the second and the second compared to the third samples is calculated as lower than the actual titre. As a result, it could be another reason that the IgM levels in the first, second, and third samples are not significantly different [15, 16].

As we move from the first samples to the third, the IgG levels in patients with different symptom severity levels (severe, moderate, and mild) significantly decreased. According to the measured IgG levels in patients with mild symptoms, it was observed that the level of IgG decreased over time, but it was not statistically significant. It could be due to the fact that in these patients, initially, the immune system functioned, well and antivirus antibodies were produced in a timely and sufficient manner. Therefore, it did not require the explosive production of antibodies. It means that from the very beginning, the immune system of these people stopped the virus and did not allow the virus to multiply and cause severe damage to the body tissues; thus, the disease did not shift towards severe symptoms. In fact, the level of antibodies was not very high at first. Therefore, the amount of IgG decreased over time in a gradual fashion. On the other hand, in patients with severe symptoms, the level of IgG was higher than in other patient groups and decreased over time significantly. As a possible explanation, the authors hypothesize that at the onset of the disease, the immune system of these patients does not stop the virus, probably due to IFN-I response impairment, so it multiplies in the body tissues widely and leads to severe clinical symptoms [17]. Thereafter, the immune system tries to produce antibodies in a burst, and the higher secreted IgG may cause more inflammation, most likely through complement activation and opsonization, than protection. In agreement with this, Long et al. showed that the IgG level in the serum of the symptomatic group was significantly higher than those in the asymptomatic group in the acute phase. Also, Long et al. showed that the IgG level in the symptomatic group was still significantly higher than those in the asymptomatic group till 8 weeks after they were discharged from the hospital [18].

Some evidence indicate that the disease severity of SARS-CoV-2 infection is associated with a dysregulated innate immune response. In innate immunity against SARS-CoV-2 infection, IFN-I plays a critical role since it inhibits viral replication in infected cells and has a defensive role in uninfected cells [17, 19]. Impairment of the IFN-I response due to the suppression of the immune system of the infected person by SARS-CoV-2 or due to the inherent weakness in the host’s innate immune system, or both, relative to the onset of symptoms probably results in high viral replication and produces an exaggerated inflammatory response including a burst but late high IgG secretion. So, in this study, it would be good if we measured not only serum IgG and IgM but also the serum type I interferons (IFN-α and IFN-β) earliest at the onset of symptoms.

## Conclusion

Although we usually expect that anti-SARS-CoV-2 antibodies have a protective role against the virus, the direct association between IgG levels and the severity of symptoms could be due to the reason that the immune system has acted late against the virus in patients with severe symptoms. Therefore, although at a later time the immune system tries to produce antibodies in a burst, the higher secreted IgG may cause more inflammation than protection. On the contrary, in patients with mild symptoms, when the immune system reacts against the virus on time, the antibodies are produced in sufficient quantities and protect the host cells from the virus infection.

## Supporting information

Akbarian et al. supplementary material Akbarian et al. supplementary material

## Data Availability

The data of the current paper are available at: https://www.cambridge.org/core/journals/epidemiology-and-infection/information/transparency-and-openness-policy.

## Supplementary material

For supplementary material accompanying this paper visit http://doi.org/10.1017/S0950268823001437. click here to view supplementary material
